# The Quinazoline Derivative, QNZ, Alleviates Experimental Autoimmune Encephalomyelitis by Suppressing Th1 and Th17 Cells

**DOI:** 10.1111/cns.70555

**Published:** 2025-08-05

**Authors:** Fan Yang, Yuan Yang, Gan Zhang, Juan Li, Shan Chen, Yan Zhou, Yuhang Kong, Xingyan Luo, Yang Liu, Ying Xu, Yantang Wang

**Affiliations:** ^1^ Clinical Laboratory Clinical Medical College and The First Affiliated Hospital of Chengdu Medical College, Chengdu Medical College Chengdu Sichuan China; ^2^ Department of Pharmacology School of Pharmacy, Chengdu Medical College Chengdu Sichuan China; ^3^ Department of Rheumatology and Immunology Clinical Medical College and The First Affiliated Hospital of Chengdu Medical College, Chengdu Medical College Chengdu Sichuan China; ^4^ Department of Emergency West China Second University Hospital, Sichuan University Chengdu Sichuan China

**Keywords:** experimental autoimmune encephalomyelitis, multiple sclerosis, neuroinflammation, QNZ, Th17 cells, Th17.1 cells

## Abstract

**Aims:**

Multiple Sclerosis (MS) is a neuroinflammatory and neurodegenerative disease affecting the central nervous system (CNS). Substantial evidence implicates a central role for CD4+ T cells in MS pathogenesis, particularly IFN‐γ+ Th1 cells and IL‐17+ Th17 cells. NF‐κB plays an essential role in regulating the differentiation of Th1 and Th17 cells, which typically mediate inflammatory responses as self‐triggers. QNZ is a highly selective inhibitor of NF‐κB transcriptional activation. In this study, we assessed the impact of QNZ on CD4+ T‐cell polarization in MS. Utilizing the experimental autoimmune encephalomyelitis (EAE) model, we investigated these aspects of MS.

**Method:**

EAE was induced in C57BL/6 female mice by active immunization with myelin oligodendrocyte glycoprotein (MOG)_35–55_ peptide. QNZ was injected intraperitoneally (*i.p*.) once every 2 days after the first immunization. Disease severity was clinically assessed and histopathologically assessed in the CNS. Phenotyping of CD4+ T cells was performed by flow cytometry in the spleen and cervical lymph nodes.

**Results:**

Prophylactic administration of QNZ to EAE mice suppressed the differentiation of Th1 and Th17 cells and demyelination within the spinal cord. Notably, QNZ also reduced the proportion of IFN‐γ+IL‐17+ Th17.1 cells, potentially playing a critical role in MS pathogenesis.

**Conclusions:**

Quinazoline derivative QNZ could suppress neuroinflammation, alleviate the progression of EAE and be associated with reduced Th1 and Th17 immunity.

## Graphical abstract

QNZ, an NF‐κB inhibitor, suppresses Th1 and Th17 cell differentiation, reduces IFN‐γ+IL‐17+ Th17.1 cells, and alleviates neuroinflammation and demyelination in an EAE mouse model of MS, highlighting its potential as a therapeutic strategy for multiple sclerosis.

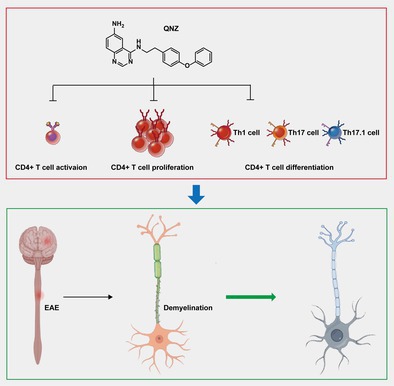

## 1 Introduction

Multiple sclerosis (MS) is a demyelinating autoimmune disease mainly caused by the infiltration of myelin‐specific, self‐reactive CD4+ T cells into the central nervous system (CNS) [1]. Relapsing–remitting MS (RR‐MS), the most prevalent form of MS, affects approximately 85% of patients. Most RR‐MS patients eventually progress to secondary progressive MS (SPMS), characterized by chronic and progressive pathological features [2, 3]. Experimental autoimmune encephalomyelitis (EAE) is a T cell‐mediated autoimmune disease model that closely replicates a significant proportion of the pathological features observed in MS, including demyelination, blood–brain barrier disruption, and the infiltration of myelin‐specific CD4+ T cells [4, 5]. Dysfunctional myelin‐specific CD4+ T cells, characterized by abnormalities in antigen recognition, activation, proliferation, differentiation, and effector functions, play a critical role in the pathogenesis of MS and EAE [6].

Myelin‐specific CD4+ T cells most commonly implicated in the development of MS belong to the Th1 and Th17 lineages, characterized by the production of interferon gamma (IFN‐γ) and interleukin 17 (IL‐17), respectively [7, 8]. IFN‐γ is a critical mediator of myelin antigen uptake by resident microglia, infiltrating peripheral macrophages, and dendritic cells during the course of MS/EAE [9]. IL‐17 stimulation of endothelial cells, astrocytes, and microglia induces the production of various cytokines and chemokines, promoting the influx of immune cells into the CNS and mediating neuroinflammation. This process also disrupts blood–brain barrier (BBB) tight junctions, mediates apoptosis, and inhibits oligodendrocyte differentiation [10, 11]. Additionally, a newly identified lineage of CD4+ T cells, termed Th17.1, characterized by the production of both IFN‐γ and IL‐17, is elevated in brain lesions and peripheral circulation in MS [7]. Th17.1 cells exhibit stronger blood–brain barrier (BBB) penetration and higher proliferation compared with Th1 or Th17 cells, and appear to be resistant to suppression by regulatory T cells [12, 13]. Dysregulation of myelin‐specific CD4+ T‐cell differentiation is closely associated with the development of MS [14].

NF‐κB signaling encompasses two primary pathways: the canonical and noncanonical pathways, both of which are crucial for the activation, differentiation, and effector/memory functions of CD4+ T cells [15, 16]. Canonical NF‐κB members, such as RelA and c‐Rel, mediate the expression of the Th17 lineage transcription factor RORγt, thereby promoting Th17 differentiation [17]. T‐cell‐specific deletion of IKKβ impairs CD4+ T‐cell activation and renders the cells resistant to the induction of Th17‐dependent EAE [18]. The noncanonical NF‐κB pathway is also essential for the pathological effector functions of Th1 and Th17 cells in neuroinflammation. Mutant mice expressing a nonprocessable form of p100 exhibit impaired generation of inflammatory CD4+ Th1 and Th17 cells [19]. Furthermore, CD4+ T cells from NF‐κB‐inducing kinase knockout mice exhibit a defective memory response upon acute viral infection [20]. Given the involvement of NF‐κB activation in CD4+ T‐cell function, targeting NF‐κB signaling represents a promising approach for anti‐inflammatory therapies.

This study focuses on a quinazoline derivative, N4‐(4‐phenoxyphenethyl)quinazoline‐4,6‐diamine (QNZ). QNZ is a highly selective NF‐κB inhibitor and has recently become a focal point in oncology research; QNZ could alleviate hepatocellular carcinoma by inhibiting the inflammatory pathway [21]. Moreover, QNZ could relieve the inhibitory effects of fibroblasts on the homing and osteogenic differentiation of mesenchymal stem cells [22]. In osteoarthritis, QNZ may protect human chondrocyte degeneration by promoting glucose uptake through Glut4 activation [23]. These studies suggest that QNZ can directly or indirectly affect the function of nonimmune cells by modulating the inflammatory response, yet there remains limited research on the regulatory effects of QNZ on immune cells in autoimmune diseases. Our previous study demonstrated that the quinazoline derivative could suppress B‐cell hyperactivation and ameliorate the severity of systemic lupus erythematosus in mice [24]. Moreover, QNZ has demonstrated neuroprotective effects and the potential to penetrate the BBB, suggesting its therapeutic potential in treating Huntington's disease [25]. However, it remains unclear whether QNZ affects pathological CD4+ T‐cell function in neuroinflammation associated with MS/EAE. This study aims to investigate the effects of QNZ on myelin‐specific CD4+ T‐cell‐mediated murine models of EAE, both in vitro and in vivo.

## 2 Materials and Methods

### 2.1 Reagents and Antibodies

Special antibodies included FITC‐conjugated anti‐CD4 (GK1.5 clone, 100,406), PE‐cy7‐conjugated anti‐CD11c (N418 clone, 117,318), PE‐conjugated anti‐IL‐17A (TC11‐18H10.1 clone, 506,904), APC‐conjugated anti‐IFN‐γ (XMG1.2 clone, 505,810), FITC‐conjugated anti‐CD83 (Michel‐19 clone, 121,506), Alexa Fluor 647‐conjugated anti‐CD80 (16‐10A1 clone, 104,718), BV650‐conjugated anti‐CD86 (GL‐1 clone, 105,035), BV421‐conjugated anti‐I‐A/I‐E(MHC‐II) (M5/114.15.2 clone, 107,631), PE‐conjugated anti‐CD69 (H1.2F3 clone, 104,508), BV421‐conjugated anti‐CD25 (PC61 clone, 102,033), PE‐conjugated anti‐CD44 (IM7 clone, 103,008), APC‐cy7‐conjugated anti‐CD62L (MEL‐14 clone, 104,428), purified antimouse CD4 (H129.19 clone, 130,302), and 5(6)‐carboxyfluorescein diacetate succinimidyl ester (CFSE) were purchased from Biolegend (San Diego, USA). The FITC Annexin V Apoptosis Detection Kit with 7‐AAD, mouse IL‐6 ELISA kit, mouse IL‐10 ELISA kit, and Perm/Wash Perm/Wash Buffer were purchased from BD PharMingen (San Diego, USA). The purified antimouse IL‐17A (TC11‐18H10.1 clone, 506,902) was purchased from Abcam (Cambridge, USA). Other special reagents included Alexa Fluor 594 goat antirabbit lgG(H+L) and Alexa Fluor 488 goat antirat lgG(H+L) were purchased from Invitrogen (Waltham, USA), and the mouse IFN‐γ ELISA kit and mouse IL‐17 ELISA kit were purchased from MABTEACH. QNZ (Selleck, Houston, USA) and FK‐506 (tacrolimus) were purchased from CNS Pharm (Houston, USA). These chemical compounds were dissolved in dimethyl sulfoxide (DMSO).

### 2.2 Co‐Cultivation of Bone Marrow‐Derived Dendritic Cells (BMDCs) and CD4+ T Cells

Female C57BL/6 mice were euthanized at 6–8 weeks. Bone marrow cells were taken from the mice and cultured in a complete medium containing 20 ng/mL of GM‐CSF (Peprotech) on a 24‐well flat plate for 8 days. Cells were collected after 2 days of stimulation with 1 μg/mL LPS (Sigma‐Aldrich). Female OT‐II mice at 6–8 weeks of age were euthanized, and their splenic CD4+ T cells were purified by negative selection using a naive CD4+ T Cell Isolation Kit (Miltenyi Biotec, Germany, 130–104‐453). Mature DCs were infused with 5 μg/mL OVA peptide_323‐339_ (ChinaPeptides) for 1 h, then collected and rinsed with PBS twice. Adjusting cell density, DCs and T cells were co‐cultured at a ratio of 1:10 with QNZ or FK‐506 in a 96‐well U‐plate in RPMI 1640 supplemented with 10% fetal bovine serum (FBS, Gibco, USA), 1% penicillin, and streptomycin (Hyclone, USA) for 72 h. Vehicle control is 0.1% DMSO diluted in PBS solution. The co‐culture system without OVA_323‐339_ was used as a blank control.

### 2.3 Flow Cytometry

The proliferation of OT‐II CD4+ T cells was analyzed for CFSE dilution by flow cytometry. The Co‐cultured cells were stained with FITC‐anti‐CD4, PE‐anti‐CD69, BV421‐anti‐CD25 and analyzed by flow cytometry. After 72 h of co‐cultivation, 10 ng/mL Phorbol 12‐myristate 13‐acetate (PMA), 1 μg/mL ION and 1 μg/mL Golgiplug (Brefeldin A, BFA) were added to cells and continued to cultivate for 4 h. The cells were stained with FITC‐anti‐CD4, APC‐anti‐IFN‐γ, and PE‐anti‐IL‐17A. Data were acquired with a Novocyte Quanteon Flow Cytometer (Agilent) and were analyzed using NovoExpress software (Agilent).

Splenic and lymph node mononuclear cells were stained with a combination of multiple mAbs against surface markers in the dark for 30 min on ice. The fluorescent mAbs for CD4+ T‐cell activation were FITC‐anti‐CD4, PE‐anti‐CD69, and BV421‐anti‐CD25. The fluorescent mAbs for CD4+ T‐cell development were FITC‐anti‐CD4, PE‐anti‐CD44, and PE‐cy7‐anti‐CD62L. The fluorescent mAbs for Th1/Th17/Th17.1 cells included FITC‐anti‐CD4, APC‐anti‐IFN‐γ, and BV421‐anti‐IL‐17A, respectively.

### 2.4 Induction and Assessment of EAE

EAE was induced in 6–8‐week‐old C57BL/6 female mice by subcutaneously injection (s.c.) of 200 μg emulsion containing MOG_35‐55_ and complete Freund's adjuvant (CFA, Chondrex, USA) (1:1 (v/v)) over four sites in the flank. Each mouse was injected with 200 ng pertussis toxin (PTX, Sigma‐Aldrich, USA) at d 0 and d 2. From Day 2, 0.2 mg/kg QNZ or 1 mg/kg FK‐506 (positive control) were intraperitoneally injected once a day for 20 d. Vehicle control is 0.1% DMSO diluted in PBS solution. There were 10 mice in the QNZ treatment group, 10 mice in the vehicle control group and 10 mice in the positive control group. Clinical scores were made according to the Benson score.

### 2.5 Histopathology and Immunofluorescence

On Day 21 postimmunization, the mice were anesthetized (2% pentobarbital sodium, 50 mg/kg), perfused with saline and 4% (w/v) paraformaldehyde. Spinal cords of mice were dissected, and embedded in paraffin. Then, pathological sections of the spinal cords were stained with hematoxylin and eosin (H&E) and luxol fast blue (LFB) (Beyotime, China). Images were captured using an OLYMPUS BX63 (Japan). The dissected spinal cords were also embedded in optimum cutting temperature (OCT) compound (Sakura, USA). After freezing, the spinal cords were stained with antimouse IL‐17A (1:200; Abcam) and antimouse CD4 (1:100; BD Pharmingen, USA, GK1.5 clone), followed by staining with Alexa Fluor 594 goat antirabbit IgG (H+L) (1:500; Invitrogen) and Alexa Fluor 488 goat antirat IgG (H+L) (1:2000; Invitrogen). Immunofluorescence was observed using a Nikon A1 Laser Scanning Confocal Microscope.

### 2.6 ELISA Assay

After 72 h co‐cultivation, supernatants were analyzed for cytokine secretion by using mouse IL‐6 ELISA kit (BD PharMingen, San Diego, USA), mouse IL‐10 ELISA kit (BD PharMingen, San Diego, USA), mouse IFN‐γ ELISA kit (MABTEACH), and mouse IL‐17 ELISA kit (MABTEACH). EAE mice were anesthetized by intraperitoneal injection of 2% pentobarbital sodium on Day 21. Bloods were collected from the eyeballs and were collected. The whole blood samples were centrifuged (1000 g, 20 min) overnight at 2°C–8°C to obtain serum. Mouse IFN‐γ and IL‐17 ELISA kit were used to detect cytokine secretion.

### 2.7 Quantitative PCR

Total RNA was extracted from the spinal cords in individual groups of mice using TRIzol reagent (Takara), following the manufacturer's instructions. Subsequently, 2 μg total RNA of each sample was reversely transcribed to cDNA using the iScript Advanced cDNA Synthesis Kit, according to the manufacturer's protocol (Bio‐Rad). The relative levels of T‐bet, ROR‐γt, and Foxp3 mRNA transcripts to GAPDH were determined by quantitative real‐time PCR (CFX 96, Bio‐Rad) using iQ SYBR Green Supermix (Bio‐Rad) and specific primers. The sequences of primers were sense 5′‐AGCAAGGACGGCGAATGTT‐3′ and antisense 5′‐GGGTGGACATATAAGCGGTTC‐3′ for T‐bet; sense 5′‐GACCCACACCTCACAAATTGA‐3′ and antisense 5′‐AGTAGGCCACATTACACTGCT‐3′ for ROR‐γt; sense 5′‐CCCATCCCCAGGAGTCTTG‐3′ and antisense 5′‐ACCATGACTAGGGGCACTGTA‐3′ for Foxp3; and sense 5′‐AGGTCGGTGTGAACGGATTTG‐3′ and antisense 5′‐TGTAGACCATGTAGTTGAGGTCA‐3′ for GAPDH. The levels of mRNA transcripts were normalized to GAPDH and analyzed by the 2^−ΔΔCt^ method.

### 2.8 Statistical Analysis

Data are expressed as mean ± SEM. Normality was assessed for all data before analysis using the Shapiro–Wilk test to test for normal distribution. The difference among groups was analyzed by repeated measures analysis of variance (ANOVA) followed by LSD post hoc test or nonparametric Kruskal–Wallis tests (non‐Gaussian distribution). Weight and mean clinical score of EAE mice were analyzed by two‐way ANOVA with Dunnett's multiple comparisons test. Graphs were plotted and data were analyzed using GraphPad Prism (version 9.0.0). Statistical significance was set at *p < 0.05, **p < 0.01.

## 3 Results

### 3.1 QNZ Efficiently Restricted Antigen‐Specific CD4+ T‐Cell Activation and Proliferation In Vitro

**FIGURE 1:**
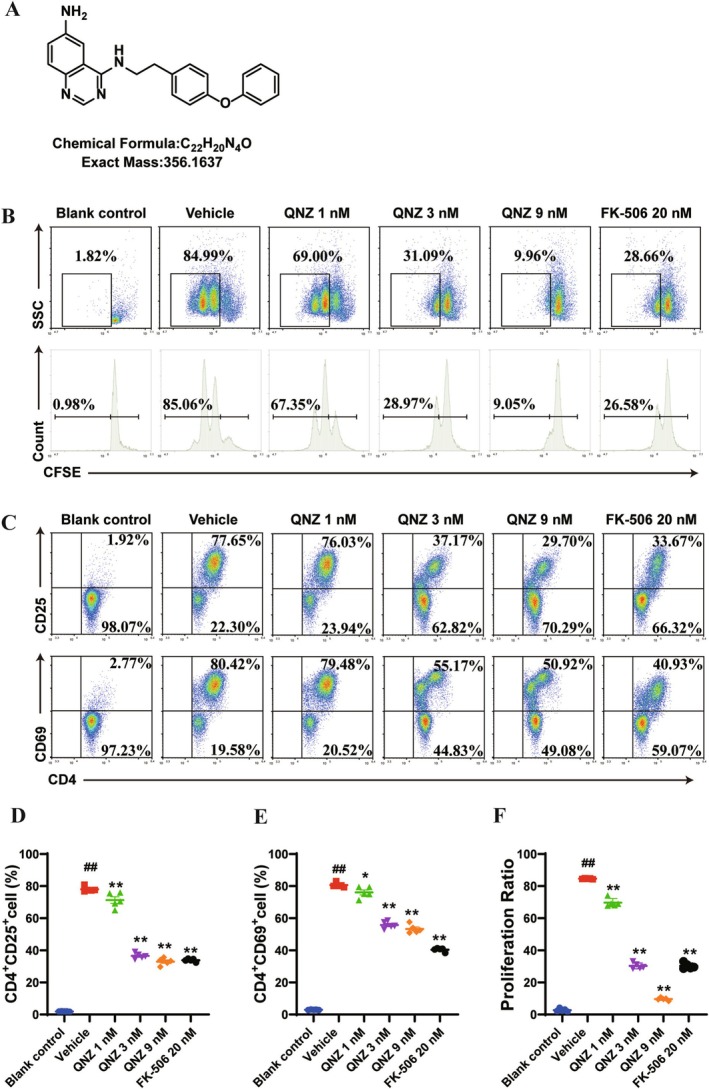
QNZ inhibited antigen‐specific CD4+ T‐cell priming in vitro. (A) The chemical structure of QNZ. (B) OT‐II CD4+ T cells and BMDCs pulsed with OVA_323‐339_ were co‐cultured with QNZ or FK‐506. The proliferation of the antigen‐specific CD4^+^ T cells was analyzed by flow cytometry after 72 h of co‐culture. (C) Flow cytometry analysis of activated CD4+ T cells. (D) Representative histograms of CFSE dilutions in CD4+ T cells. (D, E) Percentages of CD69+ and CD25+ cells on gated CD4+ T cells are shown. (F) Quantitative analysis of CD4+ T‐cell proliferation. The data are expressed as the mean ± SEM from 5 independent experiments involving 5 different mice. ^#^ *p* < 0.05, ^##^ *p* < 0.01, compared with control. *p < 0.05, **p < 0.01, versus vehicle.

Patients with MS exhibited abnormal activation and proliferation of myelin protein‐specific CD4+ T cells [26]. We initially investigated the inhibitory effect of QNZ on antigen‐specific CD4+ T‐cell activation and proliferation. To achieve this, we co‐cultured naïve CD4+ T cells from OT‐II mice with bone marrow‐derived dendritic cells (BMDCs) from C57BL/6 mice, which were pulsed with the OVA_323‐339_ peptide, following QNZ treatment. The results demonstrated that stimulation with the OVA323‐339 peptide significantly increased the activation and proliferation of OT‐II CD4+ T cells in the co‐culture system, and the levels of OT‐II CD4+ T‐cell activation and proliferation were markedly reduced by QNZ or FK‐506 treatment compared to the control group (Figure 1B,C). The inhibitory effects of QNZ on antigen‐specific CD4+ T‐cell proliferation and activation were most pronounced at medium‐to‐high concentrations (Figure 1D–F).

### 3.2 QNZ Inhibited Antigen‐Specific CD4+ T‐Cell Differentiation and Cytokine Secretion In Vitro

**FIGURE 2:**
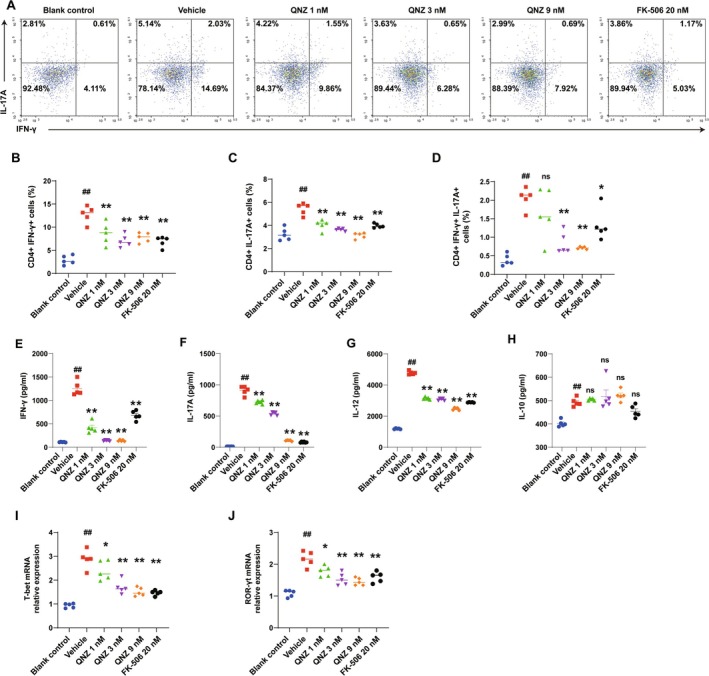
QNZ suppressed differentiation and cytokine secretion of antigen‐specific CD4+ T cells in vitro. After co‐culturing mDC (from C57BL/6 mice) and CD4+ T cells (from OT‐II mice) for 72 h, 10 ng/mL PMA, 1 μg/mL ION, and 1 μg/mL Golgiplug (BFA) were added to stimulate for 4 h, and CD4+ T‐cell differentiation in vitro was detected by flow cytometry. (A) Flow cytometry analysis of the differentiation of CD4+ T cells. (B) Th1 cell differentiation status. (C) Th17 cell differentiation status. (D) Th17.1 cell differentiation status. The co‐culture supernatants of each group were collected. The levels of IFN‐γ (E), IL‐17 (F) IL‐12 (G), and IL‐10 (H) were detected using an ELISA assay kit. Levels of T‐bet (I) and ROR‐γt (J) mRNA were analyzed by quantitative PCR. The data are expressed as the mean ± SEM from five independent experiments involving five different mice. ^##^ *p* < 0.01, compared with control. *p < 0.05, **p < 0.01, versus vehicle.

**FIGURE 3:**
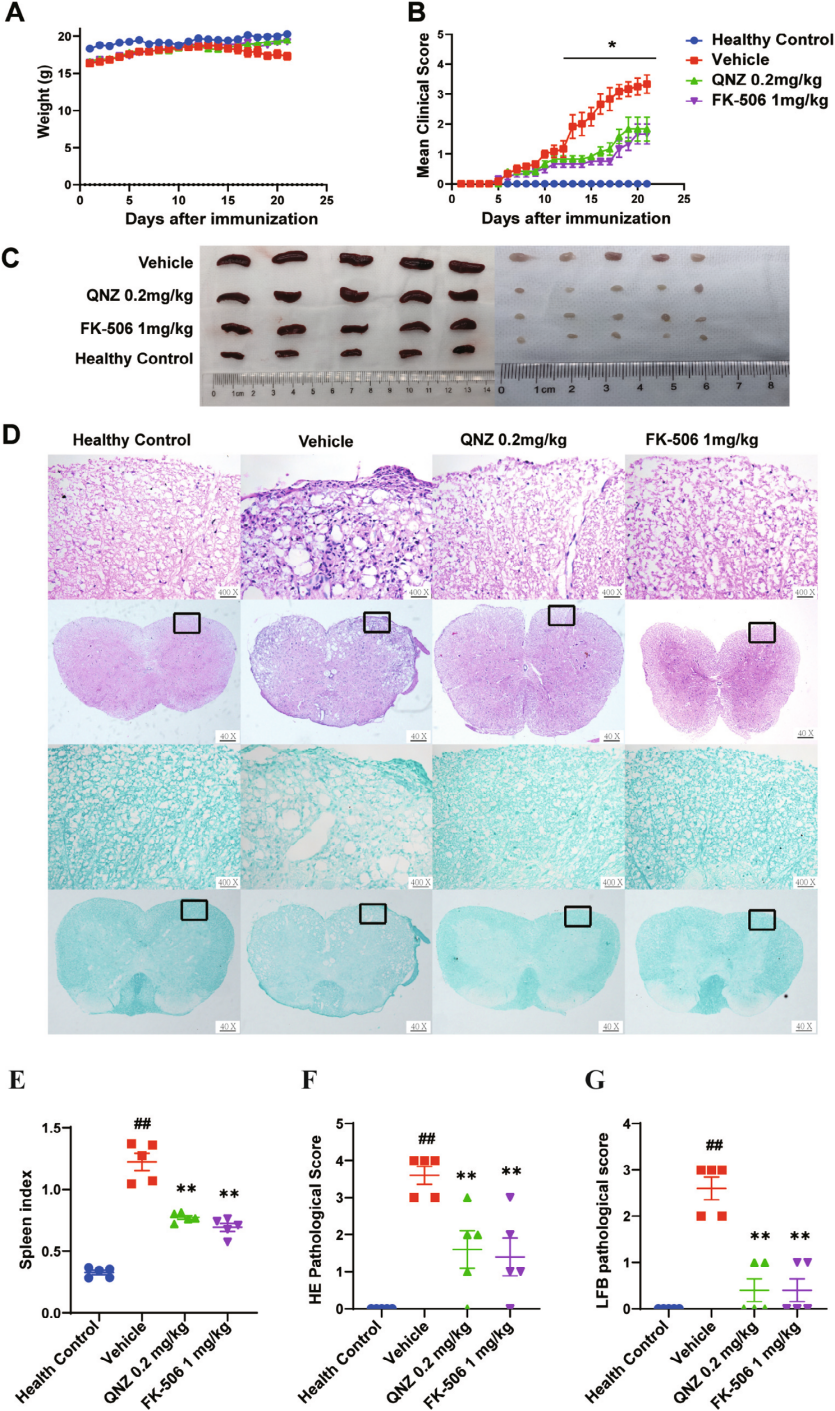
QNZ ameliorated disease progression and reduced inflammatory demyelinating lesions in EAE mice. The mouse model of EAE was induced by MOG_35‐55_ peptide immunization. QNZ and FK‐506 were intraperitoneally injected once a day for 20 d. The mice's body weight and disease score were recorded every day. On Day 21, the mice were euthanized, and their spleens and axillary lymph nodes were extracted. Pathological sections of mice spinal cords from different groups were obtained and stained with H&E and LFB. (A and B) Body weight and disease score of mice in different groups. (C) Spleen volume and lymph node size in different groups. (D) Images of spinal cords were taken at 400‐fold magnification in different groups. (E) Spleen index in different groups (Spleen index = spleen mass/body mass). (F) H&E‐stained spinal cord sections were scored on a scale of 1–5 for MOG‐induced tissue inflammation and pathology. (G) LFB‐stained spinal cord sections were scored on a scale of 1–5 for MOG‐induced tissue demyelination and pathology. Error bars represent ± SEM (*n* = 5 mice/group). ^##^ *p* < 0.01, compared with healthy control. *p < 0.05, **p < 0.01, versus vehicle.

Aberrant CD4+ T‐cell differentiation is a more significant contributor to MS or EAE pathogenesis [1]. We subsequently examined antigen‐specific CD4+ T‐cell differentiation and cytokine secretion within the co‐culture system. Compared with the vehicle group, treatment with QNZ (3, 9 nM) and FK‐506 resulted in a significant decrease in the frequencies of CD4+IFN‐γ+ Th1 and CD4+IL‐17A+ Th17 cells (Figure 2A–C). Furthermore, QNZ also reduced the proportion of CD4+IFN‐γ+IL‐17+ Th17.1 cells (Figure 2D). In MS, peripheral blood Th17.1 cells accumulate in the cerebrospinal fluid and are associated with rapidly progressive disease activity [27]. Following QNZ treatment, the secretion of proinflammatory cytokines IFN‐γ, IL‐17, and IL‐12 was significantly reduced, similar to the effect of FK‐506, compared with the vehicle group (Figure 3E–G). However, no statistically significant differences were observed in IL‐10 secretion levels among the groups (Figure 2H). Moreover, we further evaluated transcription factors associated with Th1 and Th17 populations using quantitative PCR. Our results demonstrated that the mRNA levels of T‐bet and ROR‐γt in CD4+ T cells co‐cultured with DCs were significantly reduced following QNZ treatment (Figure 2I,J).

### 3.3 QNZ Ameliorated Neuroinflammatory Demyelinating Disorder of EAE Mice

During the experimental period, mice in the healthy control group displayed good mental status and agile movements, with a consistently upward trend in body weight (Figure 3A). EAE mice in the vehicle group exhibited a gradual clumsy gait, loss of coordinated movement, paralysis in both hind and forelimbs, and body weight loss, along with mental lethargy. Mice treated with QNZ and FK‐506 showed reduced disease activity and improved mental status, compared with those in the vehicle group (Figure 3B). The spleens and lymph nodes of mice in the vehicle group showed significant enlargement compared with those of the mice in the healthy control group. After QNZ and FK‐506 treatment, the enlargement of the spleen and lymph nodes was significantly reduced in EAE mice (Figure 3C,E). On Day 21, histological examination using H&E and LFB staining was conducted on cross‐sections of the spinal cord. Histopathological findings indicated that treatment with QNZ or FK‐506 significantly reduced the infiltration of inflammatory cells and the extent of demyelination, compared with the vehicle treatment (Figure 3D,F,G).

### 3.4 QNZ Limited Excessive Activation of CD4+ T Cells In Vivo

**FIGURE 4:**
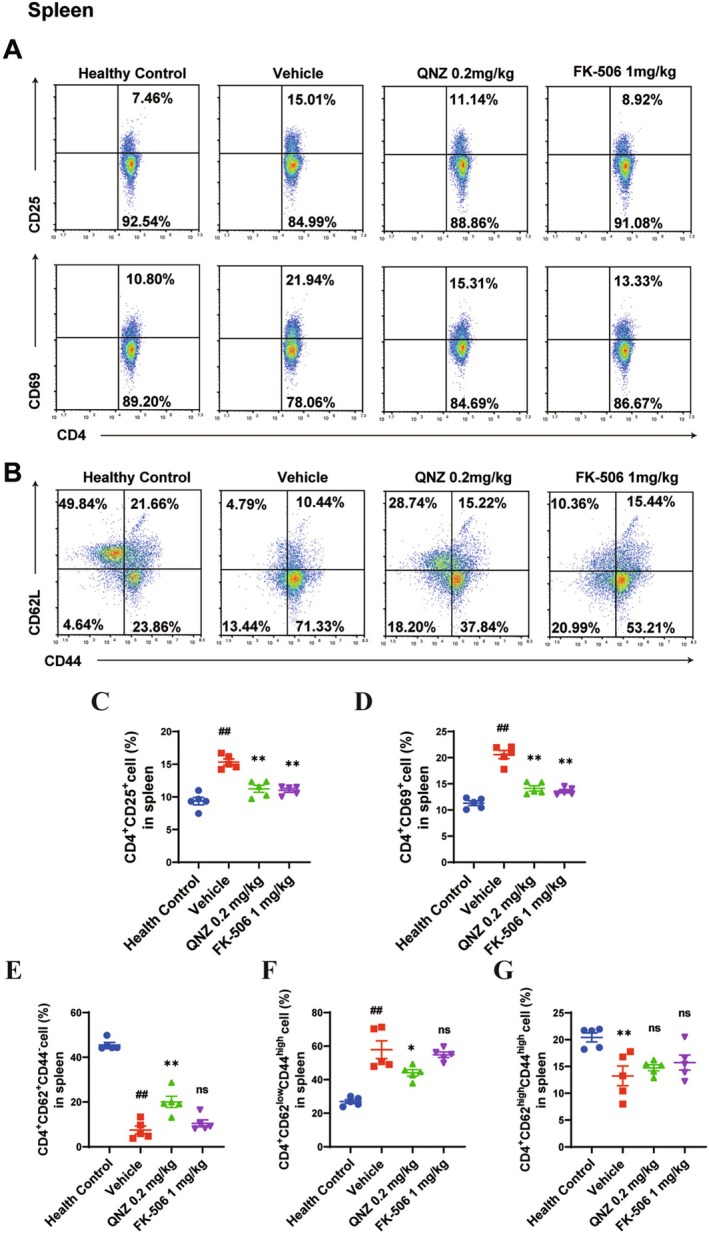
QNZ suppressed the expression of CD4+ T‐cell activation markers in vivo. On Day 21, spleens from immunized C57BL/6 mice were isolated. (A) The frequencies of CD4^+^CD25^+^ or CD4^+^CD69^+^ activated CD4+ T cells were quantified by flow cytometry. (B) The frequency of splenic naïve CD4+ T cells (CD4^+^CD62^+^CD44^−^), effector CD4+ T cells (CD4^+^CD62^low^CD44^high^), and memory CD4+ T cells (CD4^+^CD62^high^CD44^high^) was quantified by flow cytometry. (C–G) Quantitative analysis of CD4+ T‐cell subpopulation distribution within the spleens. Error bars represent ± SEM (*n* = 5 mice/group). ^##^ *p* < 0.01, compared with healthy control. *p < 0.05, **p < 0.01, versus vehicle.

On Day 21, the activation markers CD25 and CD69 of splenic CD4+ T cells from mice in each group were detected. Flow cytometry results indicated that the proportions of CD4+CD25+ and CD4+CD69+ T cells in the spleens of vehicle group mice were significantly higher than those in the healthy control group (Figure 4A). After QNZ and FK‐506 treatment, the proportions of CD4+CD25+ and CD4+CD69+ T cells in the spleens of EAE mice significantly decreased compared to the vehicle group (Figure 4C,D). At the peak of EAE, the frequency of naïve CD4+CD62L^high^CD44^low^ T cells in vehicle group mice was significantly lower, and that of effector CD4+CD62L^low^CD44^high^ T cells was significantly higher compared to the healthy control group (Figure 4B). After QNZ treatment, the frequency of naïve CD4+ T cells in vehicle group mice increased significantly, and that of effector CD4+ T cells decreased significantly compared with the vehicle group (Figure 4E,F). There was no significant difference in the frequency of memory CD4+CD62L^high^CD44^high^ T cells among these groups (Figure 4G).

### 3.5 QNZ Inhibited Pathogenic CD4+ T‐Cell Polarization in EAE Mice

**FIGURE 5:**
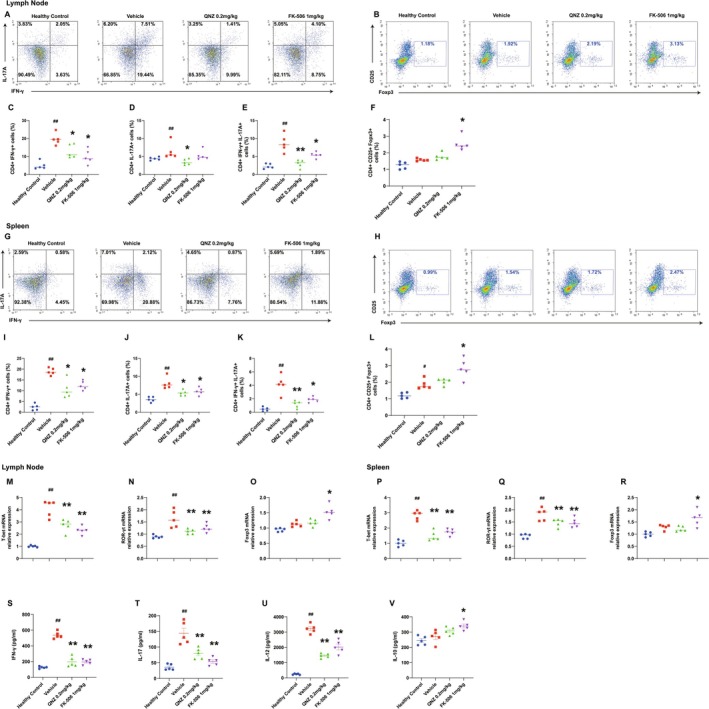
QNZ suppressed pathological CD4+ T‐cell polarization in vivo. On Day 21, spleens and lymph nodes from immunized C57BL/6 mice were isolated. (A‐B) The frequency of Th1, Th17, Th17.1, and Treg cells in the lymph nodes of EAE mice was detected by flow cytometry. (C–F) Quantitative analysis of CD4+ T‐cell subpopulation distribution within the lymph nodes. (G–H) The frequency of Th1, Th17, Th17.1, and Treg cells in the spleens of EAE mice was detected by flow cytometry. (I–L) Quantitative analysis of CD4+ T‐cell subpopulation distribution within the spleens. (M–R) Levels of T‐bet, ROR‐γt and Foxp3 mRNA were analyzed by quantitative PCR. (S–V) Serum IFN‐γ, IL‐17, IL‐12, and IL‐10 concentrations were determined using ELISA. Detection of serum cytokines IFN‐γ and IL17 using ELISA kit. Error bars represent ± SEM (*n* = 5 mice/group). ^#^ *p* < 0.05, ^##^ *p* < 0.01, compared with healthy control. *p < 0.05, **p < 0.01, versus with vehicle.

In the preceding in vitro studies, we found that QNZ inhibited antigen‐specific CD4+ T‐cell differentiation and cytokine secretion in the co‐culture system. To corroborate these findings in vivo, we next aimed to determine whether QNZ treatment suppresses pathogenic CD4+ T‐cell polarization in EAE mice. On Day 21, total mononuclear cells were obtained from the spleens and lymph nodes of healthy control, vehicle control, QNZ‐, and FK‐506‐treated EAE mice for flow cytometry analysis. ELISA was employed to assess IFN‐γ, IL‐17A, IL‐12, and IL‐10 levels in the serum of mice across different groups. The frequencies of CD4+IFN‐γ+ Th1 and CD4+IL‐17A+ Th17 cells in the spleens and lymph nodes of the vehicle group mice were significantly elevated, and the serum levels of IFN‐γ and IL‐17A were also higher compared with the healthy control group mice (Figure 5A,G). After QNZ or FK‐506 treatment, the proportions of Th1 and Th17 cells in the spleens and lymph nodes significantly decreased (Figure 5C–J), and the serum levels of IFN‐γ, IL‐17A, and IL‐12 were also reduced (Figure 5S–U) compared with the vehicle group mice. Consistent with the in vitro findings, QNZ treatment reduced the proportions of CD4+IFN‐γ+IL‐17+ Th17.1 cells in the spleens and lymph nodes of EAE mice (Figure 5E,K). However, the proportions of Treg cells in the spleens and lymph nodes, as well as the serum IL‐10 levels in EAE mice, remained unchanged following QNZ treatment (Figure 5B,H,V). Moreover, quantitative PCR results revealed that the mRNA levels of T‐bet and ROR‐γt in CD4+ T cells from the spleens and lymph nodes of EAE mice were significantly reduced following QNZ treatment (Figure 5M–Q), while Foxp3 mRNA levels remained unchanged (Figure 5O,R).

### 3.6 QNZ Decreased Th1/Th17 Cell Infiltration in the Spinal Cord of EAE Mice

**FIGURE 6:**
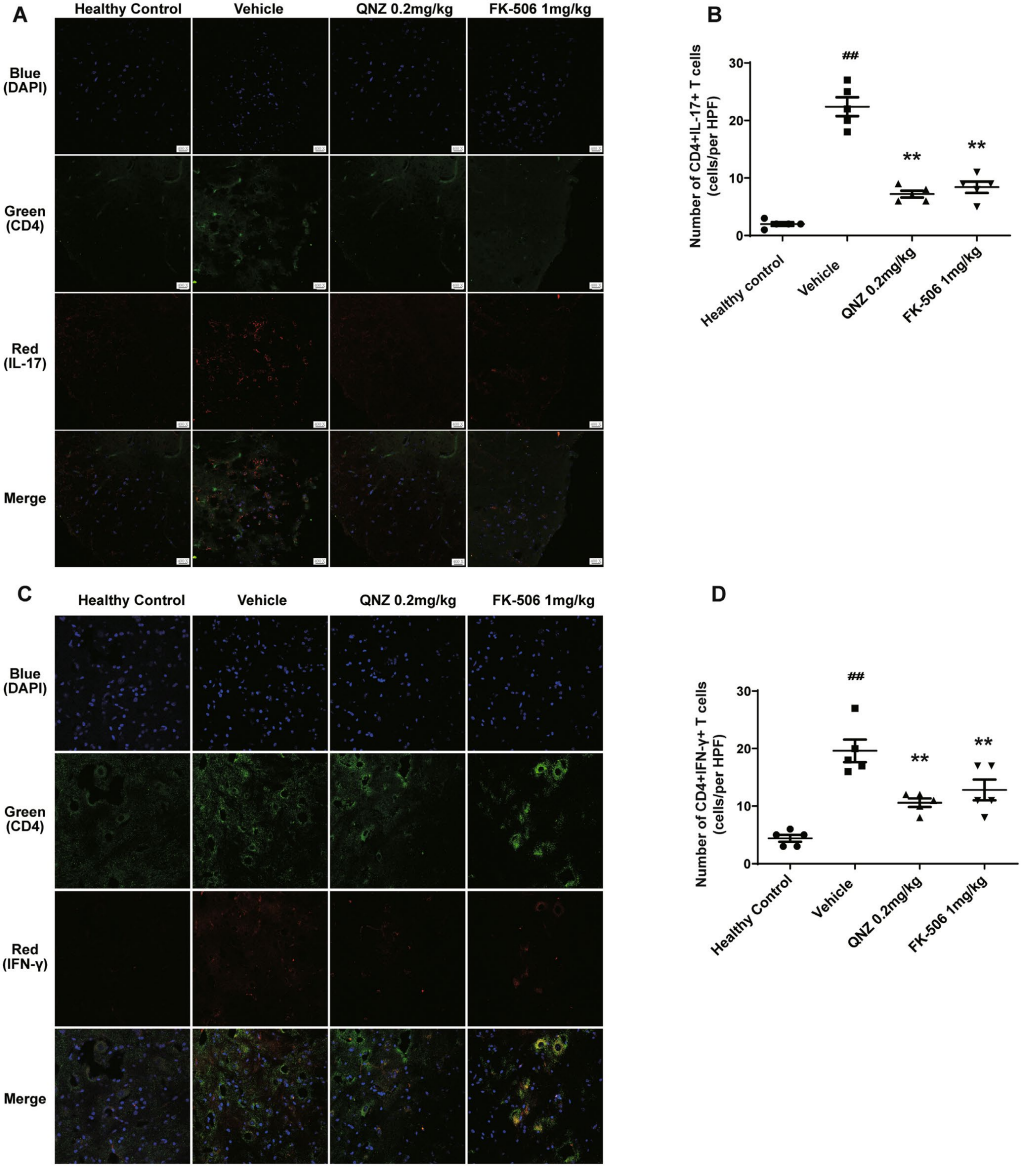
QNZ decreased Th1/Th17 cell infiltration in the spinal cords of EAE mice. On Day 21, 400‐fold magnification confocal laser scanning was used to observe the infiltration of Th1/Th17 cells into the spinal cords. Representative immunofluorescent staining pictures of spinal cords were shown. (A and C) The infiltration of Th1/Th17 cells in the sciatic nerve from different groups. (B and D) Data are expressed as numbers of positive cells per high‐powered field (HPF). Error bars represent ± SEM (*n* = 5 mice/group). ^##^ *p* < 0.01, compared with healthy control. **p < 0.01, versus vehicle.

Th1/Th17 cells play a pivotal role in MS, and our findings confirm that QNZ treatment inhibits pathogenic Th1/Th17 cell polarization in peripheral immune organs of EAE mice. Given that Th1/Th17 cells can infiltrate the central nervous system and their presence in situ is highly correlated with disease progression, immunofluorescence staining was utilized to assess Th1/Th17 cell infiltration in the spinal cords of EAE mice. As shown in Figure 6, Th1/Th17 cells in the spinal cord of the vehicle group mice demonstrated significant infiltration and considerable variation in size at 400X magnification. There was a significant decrease in the distribution of Th1/Th17 cells in the myelin sheath of the 0.2 mg/kg QNZ group and the 1 mg/kg FK‐506 group.

## 4 Discussion

Multiple sclerosis (MS) is the most prevalent autoimmune demyelinating disease affecting the CNS [1]. Aberrant activation, proliferation, and differentiation of myelin‐specific CD4+ T cells are considered key factors in the recurrence and severity of MS [28, 29]. Several therapeutic agents targeting CD4+ T cells have been developed successfully. IFNβ‐1b reduces antigen presentation and CD4+ T‐cell proliferation and is associated with a significantly lower relapse rate (31% vs. placebo) in a randomized controlled clinical trial [30]. Glatiramer acetate treatment downregulated accelerated Th1 differentiation of CD4+ T cells, reduced relapse rates, and slowed progression of disability in patients with RR‐MS [31]. Blocking repulsive guidance molecule A in EAE models suppresses Th17 cell polarization and promotes axonal regeneration, leading to improved functional outcomes [32, 33]. In this study, we found that the NF‐κB inhibitor QNZ suppresses the overactivation of CD4+ T cells both in vitro and in vivo, limits polarization towards pathogenic CD4+ T cells, and reduces the secretion of inflammatory factors, thereby alleviating the onset and progression of EAE. Following QNZ treatment, the frequencies of Th1 and Th17 cells in EAE mice decreased, serum levels of IFN‐γ and IL‐17 were reduced, and the proportion of Th17 cells decreased. Moreover, QNZ also restrains the proportion of IFN‐γ+IL‐17+ Th17.1 cells, which may play a more pivotal role in MS pathogenesis.

Previous clinical studies have confirmed the preferential transmigration of Th17.1 cells across human brain endothelial layers and their predominance in cerebrospinal fluid in patients with early MS [12]. These factors enhance the potential for Th17.1 cell infiltration into the CNS [34]. Th17.1 cells were also elevated at sites of inflammation in EAE models and were resistant to suppression by both Tregs and CTLA‐4‐Ig [35, 36]. In autoimmune encephalomyelitis, plastic Th17 cells differentiate into IFN‐γ‐producing Th17 cells (termed Th17.1 cells) driven by the inflammatory environment [34]. IL‐12 secreted by antigen‐presenting cells is essential for Th1 differentiation, and its role in the development of EAE has been extensively discussed [35]. Previous studies have confirmed that IL‐12 production by dendritic cells is regulated by NF‐κB signaling [15]. Furthermore, NF‐κB activation is indispensable for Th1 differentiation through IL‐12 [37]. As expected, the NF‐κB inhibitor QNZ significantly decreased the proportion of IFN‐γ+IL‐17+ Th17.1 cells both in vitro in an antigen‐specific co‐culture system and in vivo during EAE induction. Our results suggest that QNZ may suppress IL‐12 production by antigen‐presenting cells and dampen Th17.1 differentiation both directly and indirectly. In addition, another CD4+ T‐cell subset of interest, Tregs, exerts immunosuppressive and immune regulatory functions [38, 39]. Contrary to our expectations, vehicle‐treated mice showed a significantly higher proportion of Tregs compared with the healthy control group and the QNZ/FK‐506 group (results not shown). This discrepancy may be attributed to compensatory proliferation of Tregs in EAE mice. Thus, the alleviation of EAE in mice following QNZ treatment is likely not due to the promotion of Treg differentiation to regulate the Th17/Treg ratio balance.

The severe impact of demyelination, axonal degeneration, and neural inflammation on the quality of life of MS patients prompted this investigation. Our results revealed significant inflammatory cell infiltration, extensive demyelination, and structural damage to the myelin sheath in the vehicle group mice, while relief was observed following QNZ treatment. This effect may be attributed to weakened TCR signal transduction between myelin sheath‐specific CD4+ T cells and antigen‐presenting cells in peripheral secondary lymphoid organs, resulting from NF‐κB inhibition induced by QNZ treatment. Proliferation and activation were suppressed, leading to a higher proportion of CD4+ T cells in the treatment group being in a resting state and a subsequent reduction in effector CD4+ T cells. The reduction in Th0 cells and sustained NF‐κB inhibition led to a subsequent decrease in peripheral Th1/Th17 cells. The attenuation of peripheral Th1‐like responses modulated the overall immune system of the organism [40]. Th17 cells can disrupt the cell cycle of oligodendrocytes, impairing their survival [41]. The expression of IL‐17R in astrocytes increases under EAE conditions [42], and blocking its downstream signaling pathway improves the incidence and progression of EAE in mice [43, 44]. Our immunofluorescence results indicated a reduction in Th17 cells within the myelin sheath following QNZ treatment. This suggests that QNZ may protect the integrity of the BBB by inhibiting the Th17 cell response, thereby reducing central infiltrating cells. This inhibition contributes to the suppression of local inflammatory responses and plays a role in preserving the integrity of the myelin sheath.

It is worth noting that the NF‐κB signaling pathway is one of the most widely recognized intracellular signaling pathways in inflammatory responses, affecting not only adaptive immune cells but also innate immune cells [45]. In the context of MS/EAE, although CD4+ T cells play a dominant role in its pathogenesis, other immune cells, including B cells, macrophages, dendritic cells, and microglial cells, also influence the occurrence and progression of the disease [1]. This study focuses on the impact of QNZ on CD4+ T‐cell differentiation during the disease process, which represents both a strength and a limitation of the research. In the future, we hope to have the opportunity to employ techniques such as single‐cell RNA sequencing to comprehensively analyze the functional and phenotypic changes of different immune cell subpopulations in the context of QNZ intervention in MS/EAE. Additionally, other emerging CD4+ T‐cell subpopulations related to MS/EAE, such as Th9 and Th22 [46], also warrant further investigation.

In summary, the quinazolinone derivative QNZ effectively alleviated inflammatory demyelinating lesions in a mouse model of EAE by inhibiting the activation of antigen‐specific CD4+ T cells and suppressing the development of Th1, Th17, and Th1.17 cells. As a potential therapeutic agent for MS, QNZ warrants further investigation into its impact on different immune cell subpopulations for treating CNS autoimmune diseases and requires more comprehensive preclinical studies.

## Author Contributions

F.Y. designed and performed the experiments, collected and analyzed the data, and drafted the manuscript. Y.Y. contributed to the experimental design and revised the manuscript. G.Z. contributed to the experimental design and the manuscript. F.Y., J.L., and S.C. performed the animal experiments and collected the data. Y.Z., Y.K., and X.L. performed the in vitro experiments and revised the manuscript. Y.W., Y.X., and Y.L. designed and supervised the study and critically revised the manuscript. The authors read and approved the final manuscript.

## Ethics Statement

The study was approved by the Experimental Animal Welfare Ethics Committee of Chengdu Medical College (Approval No. CMC‐IACUC‐2021025).

## Conflicts of Interest

The authors declare no conflicts of interest.

## Data Availability

Data available from Yang, (2024), “QNZ‐EAE,” Mendeley Data, V1, doi: 10.17632/2nhwykdk9j.1 (Mendeley Data).
